# The Effect of a Mechanical Compression Device and Supraglottic Airway on Flow Time: A Simulation Study of Out-of-Hospital Cardiac Arrest in a High-Rise Building

**DOI:** 10.1155/2018/7246964

**Published:** 2018-07-16

**Authors:** Jongho Kim, Lyle Brewster, Sonja Maria, Jundong Moon

**Affiliations:** ^1^Department of Emergency Medical Services, College of Health and Nursing, Kongju National University Graduate School, Gongju, Chungnam 32588, Republic of Korea; ^2^Department of Paramedicine, School of Biomedical Sciences, Charles Sturt University, Bathurst, New South Wales 2127, Australia; ^3^Department of Emergency Medical Services, College of Health and Nursing, Kongju National University, Gongju, Chungnam 32588, Republic of Korea

## Abstract

High-rise buildings present unique challenges to providing high-quality CPR. We investigated the effect of using a mechanical compressor and supraglottic airway on flow time and CPR quality in simulated cardiac arrests occurring within a high-rise building. Twelve teams of EMS providers performed CPR according to 4 scenarios: manual compression and ventilation through bag-valve-mask (MAB) or supraglottic airway (MAS); mechanical compression and ventilation through bag-valve-mask (MEB) or supraglottic airway (MES). Chest compression indices did not differ significantly among the groups. The mechanical compression groups had a higher flow time fraction from exiting the elevator until the manikin was loaded into the ambulance than the manual compression groups. The supraglottic airway groups had higher flow time fractions from entering the elevator until the end of the scenario than the bag-valve-mask groups. The total flow time fraction was lowest in the MAB group and was highest in the MEB group (*P *< 0.001). In simulated cardiac arrest in a high-rise building, the use of a supraglottic airway maintained flow time at a level similar to that observed with the use of a mechanical compressor. Moreover, the use of a mechanical compressor and a supraglottic airway increased the flow time most effectively.

## 1. Introduction

In 2014, approximately 30,000 out-of-hospital cardiac arrests (OHCA) occurred in South Korea; 63.7% occurred at home [1], and, of those occurring at home, 60% occurred in high-rise buildings. The increasing number of people living in high-rise buildings is predicted to negatively impact OHCA survival rates, both because the response time is delayed [2] and the quality of CPR is lowered by physical challenges related to vertical evacuation in the confined space of an elevator. In a study conducted in Singapore in 2003 [3], the rates of spontaneous circulation return and survival were markedly lower among patients who suffered an OHCA in a high-rise building than at ground level. Furthermore, a Canadian study conducted in 2016 reported that the higher the floor at which the OHCA occurred, the lower the survival rate [4].

Chest compressions are essential in CPR; low-quality chest compressions are associated with insufficient coronary perfusion pressure and reduced return of spontaneous circulation and survival rate [5]. A reasonable alternative usually recommended to address the issue is the use of a mechanical compression device, which can consistently provide high-quality compressions. Although two recent large randomized controlled trials failed to demonstrate that mechanical compressions lead to a better prognosis in OHCA than manual compressions do [6, 7], they did show the low device application rate or did not calculate the down time before device application. In addition, according to the AHA guidelines for cardiopulmonary resuscitation and emergency cardiovascular care science, mechanical compressors can be used in specific settings—such as in moving ambulances or angiography suites—where high-quality manual compressions are difficult to administer [8]. High-rise buildings are another setting where the use of mechanical compressors seems suitable, especially given that the no-flow time may increase and the delivery of high-quality compressions may be impaired due to the unique challenges such buildings present; however, relevant studies on this topic are lacking.

During CPR, rescue breathing can affect the quality of chest compressions, especially flow time, although its physiological importance is lower than that of chest compressions. A simulation study conducted with lifeguards showed that mouth-to-mouth ventilation had a higher flow time than did either pocket mask or bag-valve-mask ventilation [9]. An animal study reported that coronary perfusion pressure decreases rapidly with chest compression interruptions, even those as short as the few seconds required to provide rescue breaths [10]. Advanced airways allow for administration of uninterrupted chest compressions. Of the advanced airway options, a supraglottic airway helps to maintain flow time because, unlike, with an endotracheal tube (ETT), compression pauses do not occur during its insertion.

Under these hypotheses, we performed a simulation study of the management of OHCA occurring in a high-rise building to examine the effects of mechanical compressions and supraglottic airway use on the quality of CPR, focusing on flow time using time-motion analysis.

## 2. Materials and Methods

This study was approved by the ethics committee of Kongju National University, South Korea (KNU_IRB_2015-05). Twenty-four professional EMS providers signed informed consent forms and participated in the study, conducted between 1 May and 31 July 2015.

### 2.1. Study Design and Setting

The simulation environment was created in a residential unit located in a 42-storey (130 m) high-rise building in Jeonju city, South Korea. The elevator had a 15-person capacity and travelled at 180 m/min. Its door width was 900 mm and its internal area was 1600 × 1500 mm. A LUCAS™-2 (Jolife AB, Lund, Sweden) mechanical compression device was used. This device delivers compressions at a rate of 102 (± 2) per minute with a pressure depth of 53 (± 2) mm and can be set to a 30:2 compression-to-ventilation ratio or to a continuous compression mode. A Resusci Anne® (Laerdal, Stavanger, Norway) manikin was used. Data from a monitor-defibrillator, Heartstart MRx® (Philips, Amsterdam, Netherlands), were obtained by connecting a Q-CPR measurement and feedback tool (Philips, Amsterdam, Netherlands). The suction cup of the LUCAS™-2 device was removed because it interfered with Q-CPR and the stabilisation strap was used in all cases. A Laerdal Silicone Resuscitator® (Laerdal, Stavanger, Norway) was used for bag-valve-mask ventilation and an I-gel® (Intrasurgical, Berkshire, UK) device was used for supraglottic airway ventilation. A flexible stretcher (DA-02768, Delti medical, Taiwan) was used for transport within the building.

Over a 30-min period, the participants were given the scenario description, were reminded of the CPR technique based on the 2010 American Heart Association CPR Guideline [5], and learned how to use the devices. Twelve 2-member teams were formed; an additional participant worked as an assistant for each team, preparing the elevator, carrying the monitor-defibrillator, and opening doors.

**Figure 1:**
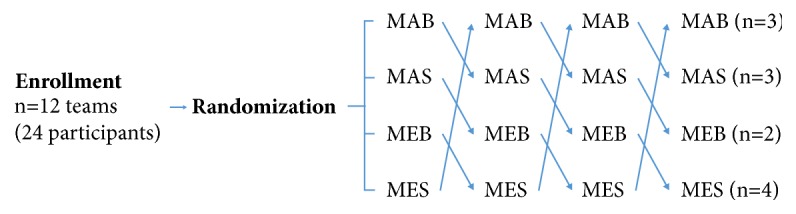
Experimental design. MAB, manual compressions with bag-valve mask ventilation; MAS, manual compression with supraglottic airway ventilation; MEB, mechanical compressions with bag-valve mask ventilation; MES, mechanical compressions with supraglottic airway ventilation.

The experiment proceeded as follows. Initially, 5 cycles of 30 compressions and 2 ventilations with direct current countershocks were performed up to 3 times, following the local EMS protocol. Then, teams were randomly assigned to 1 of the following 4 scenarios: “MAB”, administer 30 manual compressions and 2 bag-valve-mask ventilations; “MAS”, administer 30 manual compressions and 2 supraglottic airway ventilations, “MEB”, administer continuous compressions via the mechanical compression device and bag-valve-mask ventilation; and “MES”, administer continuous compressions via the mechanical compression device and supraglottic airway ventilation. Thereafter, they transported the manikin to the elevator on a flexible stretcher, exited the elevator, exited the building, and loaded the manikin into an ambulance. The next scenario began after a 20-min break. All teams performed all scenarios (Figure 1), which were video recorded and reviewed.

### 2.2. Outcome Measures

Chest compression quality was evaluated by assessing the average compression depth and rate and incomplete chest recoil ratio. Total flow time fraction and flow time fraction by phase were measured using Event Review Pro 4.2 software supplied by the monitor-defibrillator manufacturer. The duration of compression pauses and flow time fractions by phase were computed. The causes of compression pauses were identified by reviewing the video footage. The phases were defined as follows: “Phase 1”, from initiating CPR until performing CPR according to 1 of the 4 scenarios; “Phase 2”, from leaving the scene until entering the elevator; “Phase 3”, from entering until exiting the elevator; and “Phase 4”, from exiting the elevator until loading the manikin into an ambulance.

### 2.3. Statistical Analysis

Data were analyzed using IBM SPSS software, version 21.0 (Armonk, NY: IBM Corp). A significance level of* P *< 0.05 was used for all tests. Categorical variables are presented as frequencies and percentages. Continuous variables are presented as the mean ± standard deviation or median and interquartile range, as appropriate. Repeated measures ANOVA and Sidak* post hoc* tests were conducted for variables following a normal distribution based on the Shapiro-Wilk test, while the Friedman test was performed on the variables “incomplete chest recoil ratio” and “flow time fraction” because they did not follow a normal distribution. The paired* t*-test was used to examine the duration of mechanical compression device application.

## 3. Results

Of the 24 participants, 20 (83.3%) were men, 13 (54.2%) were 30–40 years old, and 20 (83.3%) had < 5 years' work experience. All were licensed BLS providers (Table 1). The average compression depth, rate, and incomplete chest recoil ratio did not differ among the groups (Table 2).

Regarding the causes of no-flow time, the time spent on rhythm analysis and defibrillator charging did not differ among the groups. The no-flow time due to artificial ventilation was significantly shorter in the supraglottic airway (MAS and MES) than in the bag-valve-mask (MAB and MEB) ventilation groups (*P *< 0.001). Interruptions due to moving the manikin were shorter in the mechanical (MEB and MES) than in the manual (MAB and MAS) compression groups (*P *< 0.001). The time taken to position and activate the mechanical compression device was similar in the MEB and MES groups.

Flow time fractions by phase were significantly higher in the manual than in the mechanical compression groups in phase 1 (*P *< 0.001). In phases 2 and 4, there was no-flow time in manual compression groups. In phases 2, 3, and 4, flow time was the highest in MES groups (*P *< 0.001).

**Figure 2:**
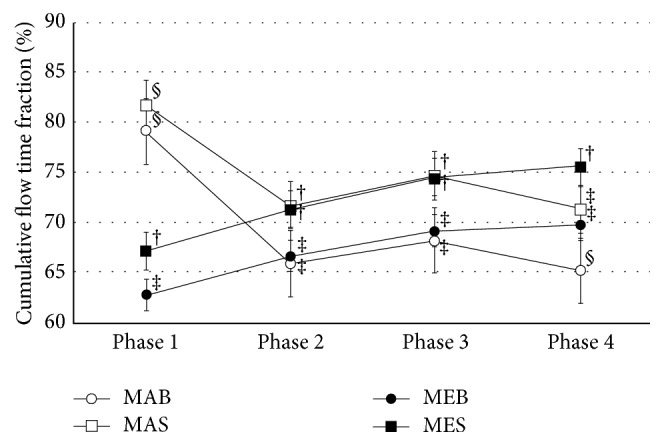
Cumulative flow time fraction in each phase. Phase 1: from initiating CPR at the scene until performing CPR according to 1 of the 4 scenarios, Phase 2: from leaving the scene until entering the elevator, Phase 3: from entering the elevator until exiting it, Phase 4: from exiting the elevator until loading the manikin into an ambulance, MAB: manual compression with bag-valve mask, MAS: manual compression with supraglottic airway, MEB: mechanical compression with bag-valve mask, MES: mechanical compression with supraglottic airway. †, ‡, § significant between group differences demonstrated in the post hoc analysis.

The comparison of cumulative flow time fraction (Figure 2) showed that, in phase 1, manual compression groups had the highest cumulative flow time fraction, but, in phases 1 through 2 and 1 through 3, supraglottic airway groups had the highest cumulative flow time fraction. Moreover, in phases 1 through 4, MES showed the highest cumulative flow time fraction, followed by MAS and MEB groups and then by the MAB group (*P *< 0.001).

## 4. Discussion

This study compared the use of a mechanical compression device or manual compressions and supraglottic airway or bag-valve-mask ventilation in a simulated OHCA scenario in a high-rise building. No significant differences were found in chest compression depth or rate and incomplete chest recoil ratio between manual and mechanical compression groups. Higher flow times were observed in the mechanical compression and supraglottic airway groups. Moreover, flow time increased most effectively in the group using both a mechanical compressor and a supraglottic airway.

The quality of manual compressions provided to a patient in a high-rise building was shown to be adequately maintained. Bloomberg et al. [11] found that a lower fraction of adequate compressions was provided by a LUCAS™ device than was provided manually; the authors proposed that this was due to the large number of cases in which the device's stabilisation strap was not used. In the present study, the stabilisation strap was used in all cases, precluding bias related to its use. However, the incomplete chest recoil ratio should be interpreted with caution as we did not use the suction cup of the LUCAS™-2 device because it interfered with the Q-CPR. However, unlike the human chest, a manikin's chest does not actively decompress; thus, even had the experiment been conducted with the suction cup in place, we believe that it would have had minimal effect on the findings.

Examined by phase, the flow time fractions in phase 1 were lowest in the mechanical compression groups due to the time taken to deploy the mechanical compressor. However, in phase 2, this interruption time was cancelled out as continuous compressions were provided even during transport. Subsequently, when using the same ventilation methods, the flow time fractions of the mechanical compression groups were similar to those of the manual compression groups and were higher in phase 4 when transport began again. In contrast, in the manual compression groups, it was almost impossible to administer chest compressions during transport. From phase 2 on, when using the same chest compression method, the supraglottic airway groups had higher flow time fractions than did the bag-valve-mask groups, as continuous compressions were provided.

The increase in flow time due to the use of a mechanical compressor during the transport of an OHCA patient is well known; however, such devices do not increase the efficacy of compressions in all situations. Levy et al. [12] reported that the proportion of total compression interruption time accounted for by deploying a mechanical compression device (LUCAS™) decreased from 18% to 10% after receiving deployment training. In the present study, the time taken to position and activate the LUCAS™-2 device was longer than that reported in the study by Levy et al. [12]. In another simulated study; Jeon et al. [13] measured flow time up to transport via ambulance to hospital approximately 5.1 km away: after performing 2 cycles of CPR at the scene and using a mechanical compression device (X-CPR™, Humed, South Korea), flow time was found to be higher when manual compression was used. Cho et al. [14] used a comparable but longer CPR scenario (5 min CPR performed at the scene and a mechanical compression device [X-CPR™] used during the 15-min ambulance transit period) and found no difference in flow time between manual and mechanical compression. Thus, the effect of a mechanical compression device on total flow time is dependent on the time taken to deploy the device and enact the protocol and implementation. Although lower in the mechanical compression groups in phase 1, the difference in flow time between the mechanical and manual compression groups disappeared after entering the elevator and remained high in the mechanical compression groups until the manikin was loaded into the ambulance. We did not measure the flow time during the ambulance transport, but we would expect the mechanical compression group to have a higher flow time afterward, based on the results of previous studies [15, 16]. Therefore, in addition to early deploying a mechanical compression device, the effect of mechanical compression on flow time should be determined by examining interruptions using time-motion analysis, according to the environment in which the OHCA occurs. Another important variable is whether the mechanical compression device is brought to the scene or is left in the ambulance to be used during transportation. This factor was considered in a previous article [17] but was not examined in the present study.

Ventilation influences flow time. Supraglottic airways do not interrupt chest compressions during their insertion and allow for maintenance of continuous compressions regardless of ventilation—their use is therefore helpful in maintaining flow time. Kurz et al. [18] reported that chest compression fractions were higher before and after insertion of a supraglottic airway than fractions following ETT in OHCA patients. However, they reported on data obtained only for 2 min before and, after insertion of an ETT, thus the difference between the groups was not large. The present study showed that the supraglottic airway groups maintained higher flow times than did the bag-valve-mask ventilation groups from phase 2 on. The finding of no difference in final flow time fraction between the supraglottic airway and mechanical compression group is noteworthy, as supraglottic airways are easier to carry and use than are mechanical compression devices.

*Limitations. *This simulation study has some limitations. First, it used a manikin; manikins differ from the human body in terms of weight, range of limb motion, skin texture, etc. The lower weight and slippery texture of the manikin's skin can affect the stable operation of a mechanical compression device. Hence, caution is required when interpreting the findings. However, during the course of the experiment, the mechanical compressor slipped on very few occasions, the chest compression indices were within the recommended ranges, and the chest recoil index was adequate despite the absence of the device's suction cup. Second, the internal environment of high-rise buildings varies. In this study, specifically, the high-rise building used had no residents as they had not yet moved in; the presence of residents could negatively affect flow time. Third, each team comprised 2 EMS providers, and an additional participant worked as an assistant for all teams. Hence, there were avoidable compression pauses during CPR, such as being unable to apply chest compressions while deploying the mechanical compression device. Flow time would have increased if teams of ≥ 3 EMS staff had provided CPR. Fourth, the participants received only 30 minutes of short training, which would have affected the LUCAS™-2 deployment time. In fact, the deployment times for LUCAS™-2 device in the MEB and MES groups were 62.6 s and 55.4 s, respectively, which is much longer compared with the values reported by Levy et al. [12], who found that team training reduced deployment time from 21 s to 7 s. Therefore, it is expected that a higher flow time can be maintained through proper training. Finally, given the small sample size, a randomized, cross-over design was not used. When we set an error probability of 0.05, and an effect size of 0.25, the sample size was 36. The number of local EMS providers was only about 110, which made it difficult to recruit more voluntary participants.

## 5. Conclusion

This study suggests that the quality of manual compressions can be adequately maintained when providing CPR to an OHCA patient in a high-rise building. The combination of manual compressions and bag-valve-mask ventilation was associated with poor flow times. Flow times were similar in the supraglottic airway and mechanical compression device groups, and the use of both a mechanical compressor and a supraglottic airway increased the flow time most effectively. This preliminary study could positively impact the emergency care given to OHCA patients in high-rise buildings. Further research is required to substantiate these findings.

## Figures and Tables

**Table 1:** Characteristics of the 24 professional EMS providers who participated in the study.

**Characteristic**	**Category**	**n (**%**)**
Sex	Male	20 (83.3)
	Female	4 (16.7)
Age, years	< 30	9 (37.5)
	30–40	13 (54.2)
	≥ 41	2 (8.3)
EMS provider career, years	< 5	20 (83.3)
	5–10	2 (8.3)
	≥ 11	2 (8.3)
Basic medical education	First level emergency medical technician	9 (37.5)
	Second level emergency medical technician	12 (50)
	Ambulance nurse	3 (12.5)
BLS provider certification	Yes	24 (100)
	No	0 (0)

**Table 2:** Comparison of chest compression indices and flow times.

	**Manual compression groups**		**Mechanical compression groups**		***p***
	**MAB**	**MAS**	**MEB**	**MES**	
**Chest compression index:**					
Compression depth (mm)^†^	57.2 ± 2.4	56.8 ± 2.9	56.0 ± 2.0	55.1 ± 2.2	0.41
Compression rate (n/min) ^†^	113.6 ± 4.6	113.7 ± 3.6	111.3 ± 2.0	111.0 ± 1.9	0.21
Incomplete chest recoil ratio (%)^‡^	5.0 (4.3–9.3)	6.0 (3.3–8.8)	11.0 (8.3–16.8)	8.5 (6.0–10.8)	0.47
Flow time fraction (%)^‡^	66.5 (61.7–67.3)^ †^	72.1 (69.2–72.7)^ ‡^	70.5 (67.6–71.8)^ ‡^	75.5 (73.9–76.8)^ §^	<0.001
Flow time (s)^†^	290.3 ± 13.3^ †^	332.2 ± 27.1^ ‡^	348.6 ± 12.0^ ‡^	378.2 ± 16.5^ §^	<0.001
Scenario duration (s)^†^	446.8 ± 22.4^ †^	472.9 ± 30.6^ †^	500.6 ± 24.9^ ‡^	501.3 ± 20.7^ ‡^	<0.001
**Activity during no-flow time: **					
Rhythm analysis and defibrillator charging (s)^†^	13.9 ± 5.3	15.4 ± 3.5	12.4 ± 3.7	11.6 ± 4.5	0.10
Artificial ventilation (s)^†^	66.2 ± 12.6 ^‡^	49.6 ± 6.0 ^†^	73.6 ± 9.2^ §^	52.8 ± 7.9^ †^	<0.001
Moving the manikin (s)^†^	76.8 ± 14.7^ ‡^	70.2 ± 10.9^ ‡^	3.4 ± 4.3^ †^	3.3 ± 7.9^ †^	<0.001
Mechanical compressor deployment (s)^†^	Not applicable	Not applicable	62.6 ± 9.0	55.4 ± 12.8	0.15
**Flow time fraction by phase:**					
Phase 1 (%)^‡^	80.5 (76.6–81.7)^ §^	81.4 (80.3–83.6)^ §^	63.5 (59.6–65.4)^ †^	67.1 (64.9–68.8)^ ‡^	<0.001
Phase 2 (%)^‡^	0.0 (0–0)^ †^	0.0 (0–0)^ †^	91.9 (86.3–90.1)^ ‡^	100.0 (96.33–100.0)^ §^	<0.001
Phase 3 (%)^‡^	84.6 (82.9–86.6)^ †^	98.4 (98.1–100.0)^ ‡^	88.5 (86.3–90.1)^ †^	100.0 (100.0–100.0)^ §^	<0.001
Phase 4 (%)^‡^	0.0 (0–0) ^†^	0.0 (0–0)^ †^	84.2 (80.0–88.7)^ ‡^	100.0 (100.0–100.0)^ §^	<0.001

Data are presented as the mean ± standard deviation or median (interquartile range). MAB, manual compression with bag-valve mask; MAS, manual compression with supraglottic airway; MEB, mechanical compression with bag-valve mask; MES, mechanical compression with supraglottic airway. ^†, ‡, §^ Significant between group differences demonstrated in the *post hoc* analysis.

## Data Availability

The data used to support the findings of this study are included within the article.
